# Historical and contemporary management of infantile hemangiomas: a single-center experience

**DOI:** 10.3389/fphar.2024.1280948

**Published:** 2024-02-02

**Authors:** Yun Zou, Zhiping Wu, Pingliang Jin, Ronghua Fu, Jun Cheng, Hanxiang Bai, Mengyu Huang, Xiangqun Huang, Hua Yuan

**Affiliations:** Department of Plastic Surgery, Jiangxi Provincial Children’s Hospital, Nanchang, China

**Keywords:** infantile hemangioma (IH), propranolol, vascular tissue, neoplasms, B-blocker

## Abstract

**Objective:** This study explores the 22-year evolution of Infantile Hemangiomas (IHs) treatment strategies at a single-center hospital, aiming to establish an individualized IHs management protocol.

**Methods:** Retrospective review of IHs infants 2000–2022 at the Department of Plastic Surgery, Jiangxi Provincial Children’s Hospital.

**Results:** In our study of 27,513 IHs cases, 72.2% were female, with the median age at first hospital visit being 25 days. The majority of cases had localized and superficial lesions primarily on the head, face, and neck (67.5%). Ulceration rates fell from 21.1% to 12.6% with the introduction of propranolol. Management strategies have shifted over time, with the proportion of cases undergoing expectant management dropping from 32.9% to 12.4%. Since 2008, 26.1% of patients were treated with oral propranolol, largely replacing corticosteroids. Topical *β*-blockers have been used in 12.1% of cases, leading to a reduction in local injection therapy from 20.8% to 13.2%. Laser therapy, introduced in 2016, has been used in 13.8% of cases, while surgical excision has dropped from 25.0% to 8.5% due to alternative treatment options. Combination therapy was used in 8.8% of cases post-2015, indicating a rising trend. Drawing from the evolution of IHs management strategies, an individualized protocol for the management of IHs was successfully established.

**Conclusion:** Treatment for IHs has evolved over recent decades, with less invasive medical interventions increasingly replacing more invasive methods. Furthermore, a personalized treatment protocol established in this study could boost the cure rate of IHs while minimizing potential side effects and complications.

## 1 Introduction

Infantile hemangiomas (IHs) are common benign vasculogenic tumors in infancy, characterized by the abnormal proliferation of vascular endothelial cells (Chen et al., 2013). The incidence of IHs is between 4% and 10%, with Caucasians and females more susceptible (Léauté-Labrèze et al., 2017; Rodríguez Bandera et al., 2021; Sebaratnam et al., 2021; Macca et al., 2022). The currently known risk factors for IHs include premature birth, low bodyweight, decreasing gestational age, miscarriage history, anemia in pregnancy, threatened miscarriage and intrauterine complications such as pre-eclampsia or placental previa, preterm premature rupture of membranes (PPROM), threatened miscarriage, premature rupture of membranes (PROM) as well as a family history of IHs (Munden et al., 2014; Goelz and Poets, 2015; Auger et al., 2017; Hunjan et al., 2017; GongXQiuTFengL et al., 2022). IHs typically manifest as solitary and superficial lesions that can occur on any part of the body, including the derma, mucous layers, and viscera, though they are most frequently found in the head-face-neck region (Satterfield and Chambers, 2019). Pathophysiology is not completely clear, it is thought to involve an imbalance in angiogenesis, potentially due to hypoxia (Colonna et al., 2010; Drolet and Frieden, 2010; de Jong et al., 2016; Léauté-Labrèze et al., 2017).

IHs typically emerge at birth or shortly after and proliferate during the first year, though initial signs may be pale areas or red macula with telangiectasia at birth or early days of life (Chang et al., 2008; Tollefson and Frieden, 2012; Léauté-Labrèze et al., 2017). Following the proliferative phase, IHs typically enter a spontaneous involution phase that starts around the first year and can last for several years (Bauland et al., 2011; Baselga et al., 2016). However, about 70% of infants experience residual effects, such as telangiectasia, excessive fibrofatty tissue, and skin laxity, and roughly 10% face complications including obstruction, functional impairment, ulceration, and disfigurement (Léauté-Labrèze et al., 2017; Gomes et al., 2023). These situations necessitate early expert consultation and intervention to prevent IHs progression.

Historically, there are numerous management methods for IHs, such as intralesional and systemic corticosteroids, chemotherapy drugs, laser therapy and surgical excision (Darrow et al., 2015). However, in 2008, propranolol was unexpectedly discovered to promote the involution of IHs when it was used to treat tachycardia in a patient with IHs (Léauté-Labrèze et al., 2008). Since then, oral propranolol has become a mainstream treatment for IHs worldwide. Numerous studies have confirmed that propranolol can inhibit proliferation and induce involution of IHs, proving more effective and safer than other treatments such as corticosteroids (Léauté-Labrèze et al., 2015; Polites et al., 2019; You et al., 2021). Despite this, treatment should still be tailored to individual patient needs, considering factors like IHs characteristics, potential complications, and patient age. Other management options may be applicable in suitable cases, especially as various treatment methods have improved in recent years. For instance, small IHs in non-critical areas (such as extremity and trunk) can usually be treated through topical timolol or laser therapy. Over the last 20 years, IHs treatment has shifted towards early intervention with appropriate methods to control growth and promote regression. Additionally, in clinical practice, a pressing need exists for tailored treatment approaches based on the unique requirements of each child’s condition, aiming to elevate the cure rate of IHs while mitigating the incidence of side effects or complications.

We previously reported the efficacy and safety of treating IHs with oral propranolol in 2023 (Fu et al., 2023) and we now report our 22-year treatment experience with IHs. This study’s aim was to describe the evolution of IHs management strategies at a single-center hospital over a 22-year period. Meanwhile, we hope to establish an individualized protocol from our previous treatment experience, which may offer the ability to refine the current treatment modality in IHs.

## 2 Materials and methods

### 2.1 Study design and sample

We performed an observational retrospective study to descript and analyze the characteristics and management of IHs in pediatric patients. We included all patients diagnosed with IHs who were followed up at the outpatient of Department of Plastic Surgery, Jiangxi Provincial Children’s Hospital were included. The inclusion criteria were infants with diagnosis of IHs and available clinical data. The exclusion criteria were patients with pathological examination showing other diagnoses after surgical removal.

Our primary objective focused on analyzing the evolution of IHs treatment methods over time. The secondary objective centered on establishing an individualized protocol aimed at enhancing the IHs cure rate while mitigating the incidence of side effects or complications.

### 2.2 Data collection and variable definition

A retrospective analysis was conducted on the prospectively-maintained database of IHs patient within the Department of Plastic Surgery, Jiangxi Provincial Children’s Hospital, spanning the period from 2,000 to 2,022. Data on demographics including sex, gestational age, age at first hospital visit and birth weight were collected. The age at first hospital visit refers to the age at which the infant, referred to our institution, was first seen.

In addition to demographic data, we also documented the characteristics of IHs, including their location, morphological subtype, and complications. IHs were categorized as superficial, deep, or combined, based on the depth of the involved tissue.

Classification by morphologic subtype included localized (grown from a single focal point or localized to an area without any apparent linear or metameric configuration), segmental (located at a recognizable and/or significant portion of a developmental segment or involving a broad anatomic territory of skin), indeterminate (neither localized nor segmental), multifocal (≥5 IHs), which was basically according to the criteria reported by Haggstrom et al., in 2005 (Haggstrom et al., 2006a). The process of diagnostic confirmation and/or exclusion of other types of IHs, as well as the necessity for expert evaluation, were also evaluated. We classified treatment approaches as follows: expectant management alone; systemic treatment options; local treatment options; laser therapy; surgical excision; and combination therapy. Expectant management alone involved lesion monitoring via ultrasound (US) and periodic clinical reviews. Systemic treatment options referred to oral administration of corticosteroids or propranolol. Local treatment options included the use of local injections of anti-tumor agents like pingyangmycin, sclerosants such as lauromacrogol, and the application of topical medications, primarily consisting of *β*-blockers. Laser therapy utilized CO_2_ laser, pulsed dye laser (PDL) with 595 wavelength or neodymium-doped yttrium aluminium garnet (ND:YAG) laser. Combination therapy was the use of multiple treatments from the above categories, such as laser therapy combined with topical *β*-blockers, local injections coupled with laser therapy or topical *β*-blockers, or oral propranolol administered alongside laser therapy or local injections.

The treatment response, which was based on clinical examination and imaging findings after treatment. IHs relapse was defined as increased IHs color, surface/volume, or texture after treatment cessation. The regression was assessed based on the reduction in the dimensions of lesions. A total regression was defined as an improvement rate that equals or exceeds 90%.

### 2.3 Ethics

This study was conducted in accordance with all relevant national regulations, institutional policies, and the principles of the Declaration of Helsinki. The research also received approval from the Institutional Review Committee of the Ethics Committee at Jiangxi Provincial Children’s Hospital.

### 2.4 Statistical analysis

Statistical analysis was carried out using IBM^®^ SPSS^®^ Statistics 27.0. Continuous variables are presented as the median and interquartile range (IQR), while categorical data are represented as the number and percentage (N, %).

## 3 Results

### 3.1 Patient demographics and hemangioma characteristics

Our study covered 27,513 infants with infantile hemangiomas (IHs) from 2000 to 2022, with a female-to-male ratio of 2.5:1. An overview of the patients and IHs is presented in Table 1. On average, their first hospital visit was at 25 days of life (interquartile range, 18–62 days), and the median birth weight was 3.2 kg (interquartile range, 1.8–5.2 kg). Premature infants comprised 31.0% (*n* = 8,534) of the patients, while the remaining 69.0% (*n* = 18,979) were term born. The IHs were most frequently located in the head, face, and neck (*n* = 18,571; 67.5%), followed by extremities (*n* = 5,089; 18.5%) and trunk (*n* = 3,853; 14.0%). Regarding the morphological subtype, 13,261 (48.2%) patients presented with localized IHs. Superficial IHs (*n* = 17,140; 62.3%) were the most common classification, followed by combined (*n* = 6,907; 25.1%) and deep IHs (*n* = 3,466; 12.6%). Complications included ulceration in 14.6% (*n* = 4,010) of patients and bleeding in 2.3% (*n* = 644).

**TABLE 1:** Baseline characteristics of patients (*n* = 27513) and IHs. IHs, infantile hemangiomas.

Characteristics	Value (%)
Patients	
Gender^a^	
Male	7964 (27.8)
Female	19549 (72.2)
Gestational age^a^	
Term born (≥37 weeks)	18979 (69.0)
Born prematurely (<37 weeks)	8534 (31.0)
Age at first hospital visit (corrected actual age, day)**	25 (18–62)
Birth weight (kg)^b^	3.2 (1.8–5.2)
**IHs**	
Location^a^	
Head, face and neck	18571 (67.5)
Extremity	5089 (18.5)
Trunk	3853 (14.0)
Morphologic subtype^a^	
Localized	13261 (48.2)
Segmental	8776 (31.9)
Indeterminate	5117 (18.6)
Multifocal	359 (1.3)
Classification^a^	
Superficial	17140 (62.3)
Deep	3466 (12.6)
Combined	6907 (25.1)
Complications^a^	
Ulceration	4010 (14.6)
Bleeding	644 (2.3)
Associated structural anomalies^a^	
PHACE syndrome	45 (0.16)
LUMBAR syndrome	12 (0.04)

^a^ Values are presented as a number (percentage).

^b^ Values are presented as a median (interquartile range).

### 3.2 Incidence and ulceration rates by historical period

**FIGURE 1:**
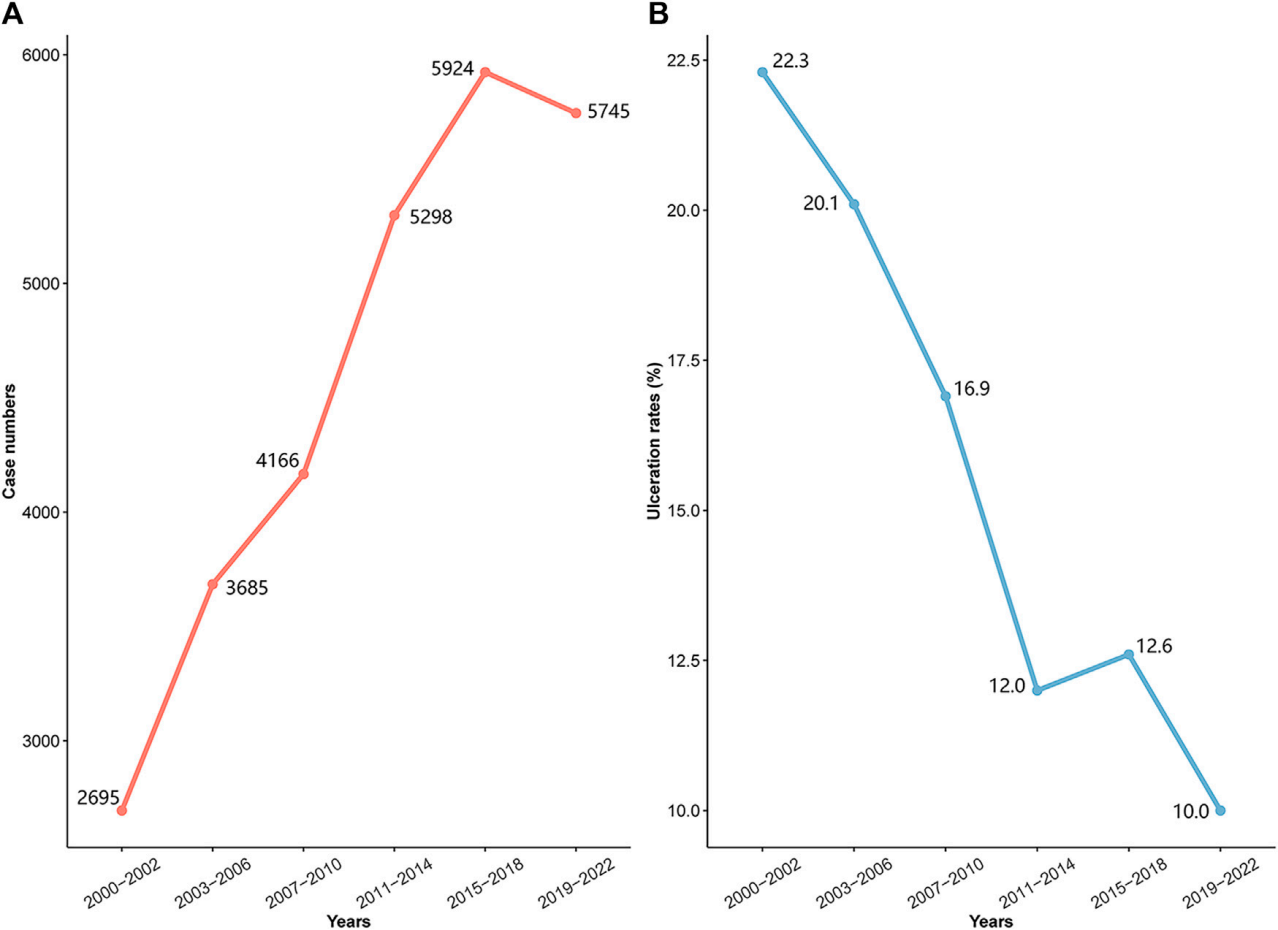
Incidence and ulceration rates by historical period; **(A)** incidence trend; **(B)** ulceration rates trend.

Figure 1A shows a significant rise in the incidence of diagnosed IHs in infants recently. However, Figure 1B shows a steady decline in ulceration rates which refers to the occurrence during treatment and at the time of visit, from 22.3% to 10.0%. A significant drop in ulceration was noted in the propranolol era with rates falling from 21.1% (1,344 out of 6,380 patients) prior to its use, to 12.6% (2,666 out of 21,133 patients).

### 3.3 Management

**FIGURE 2:**
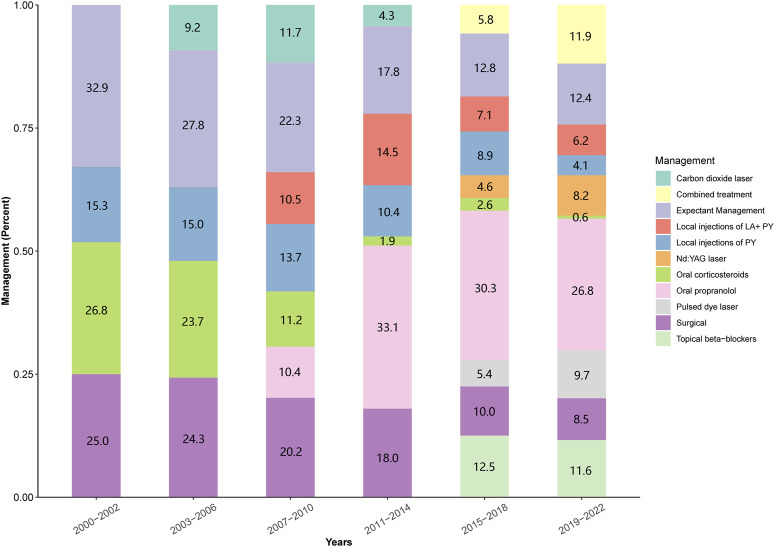
Changes in patient management by historical period; LA: lauromacrogol; PY: pingyangmycin; ND:YAG: neodymium-doped yttrium aluminium garnet.

**FIGURE 3:**
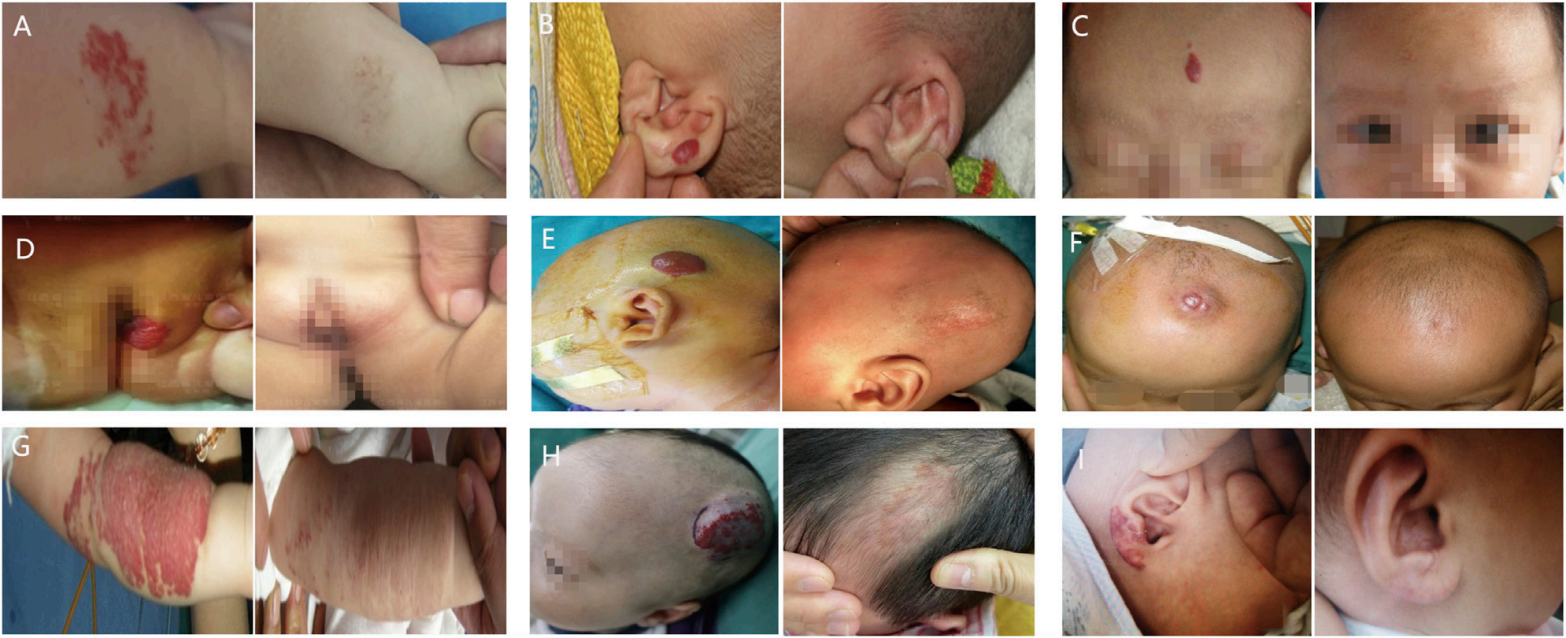
Representative clinical outcomes of each treatment options. Infantile hemangiomas (IHs) are shown before (left column), and after (right column) treatment. **(A)** IHs of right forearm treated with expectant management; **(B)** IHs of left outer auricle treated with laser therapy; **(C)** IHs of forehead treated with topical β-blockers; **(D)** IHs of the vulva treated with oral propranolol; **(E)** IHs of head treated with surgical excision; **(F)** IHs of head treated with local injections; **(G)** IHs of left forearm treated with oral propranolol combined with laser therapy; **(H)** IHs of head treated with oral propranolol combined with local injections; **(I)** IHs of right outer auricle treated with topical β-blockers combined with laser.

Figure 2 illustrates how management strategies for IHs, including expectant management, systemic and local treatments, laser therapy, surgical excision, and combination therapy, varied based on symptoms and treatment era. Representative clinical outcomes are depicted in Figure 3.

#### 3.3.1 Expectant management

Our study shows that 19.0% (5,253 out of 27,513) of patients underwent only expectant management, involving US surveillance and regular reviews, with 78.9% (4,144) having superficial IHs. As Figure 2 shows, the usage of this management method has declined from 32.9% to 12.4% over the past 2 decades, largely due to the rise of beta-blocker and laser therapies.

#### 3.3.2 Systemic treatment options

Before propranolol’s introduction in 2008, 25.0% (1,595 out of 6,380) of infants were treated with oral corticosteroids. Post-propranolol, 29.6% (6,278 out of 21,133) received systemic treatment, with 26.1% (5,522 out of 21,133) treated with propranolol, and 3.5% (756 out of 21,133) with corticosteroids. As Figure 2 shows, corticosteroid use sharply fell post-2008 and was recently only used in 0.6% (34 out of 5,745) of cases where propranolol was contraindicated or ineffective.

#### 3.3.3 Local treatment options

Local treatments, crucial for superficial IHs, were given to 20.8% (3,292 out of 15,844) of patients via injections of pingyangmycin or a pingyangmycin-lauromacrogol combo before topical *β*-blockers. Post *β*-blockers, 12.1% (1,407 out of 11,669) received topical *β*-blockers, and 13.2% (1,540 out of 11,669) had local injections. As Figure 2 shows, local injection use slightly decreased after introducing topical *β*-blocker therapy.

#### 3.3.4 Laser therapy

Since the introduction of laser equipment in our department in 2016, 1,621 out of 11,669 infants with IHs (13.8%) received laser therapy. Among these, 877 infants (7.5%) underwent 595 nm PDL therapy, while 744 infants (6.3%) received ND:YAG laser therapy. As shown in Figure 2, the use of laser therapy in treating IHs has been on an upward trend in recent years.

#### 3.3.5 Surgical excision

In our study, surgical excision was performed on 5,254 out of 27,513 patients (19.1%), and the majority of these IHs were located on the extremities or trunk (4,056 out of 5,254, 77.2%). As shown in Figure 2, the application of surgical excision has gradually decreased with the development of other treatment options, from 25.0% (674 out of 2,695) to 8.5% (488 out of 5,745).

#### 3.3.6 Combination therapy

In recent years, combination therapy has played a crucial role in enhancing the effectiveness of treatments for complex IHs, especially as various treatment methods continue to be refined. In our department, 1,027 out of 11,669 patients (8.8%) received combination therapy after 2015. Based on our experience and as depicted in Figure 2, the use of combination therapy has shown an increasing trend in recent years.

### 3.4 Individualized protocol establishment

**FIGURE 4:**
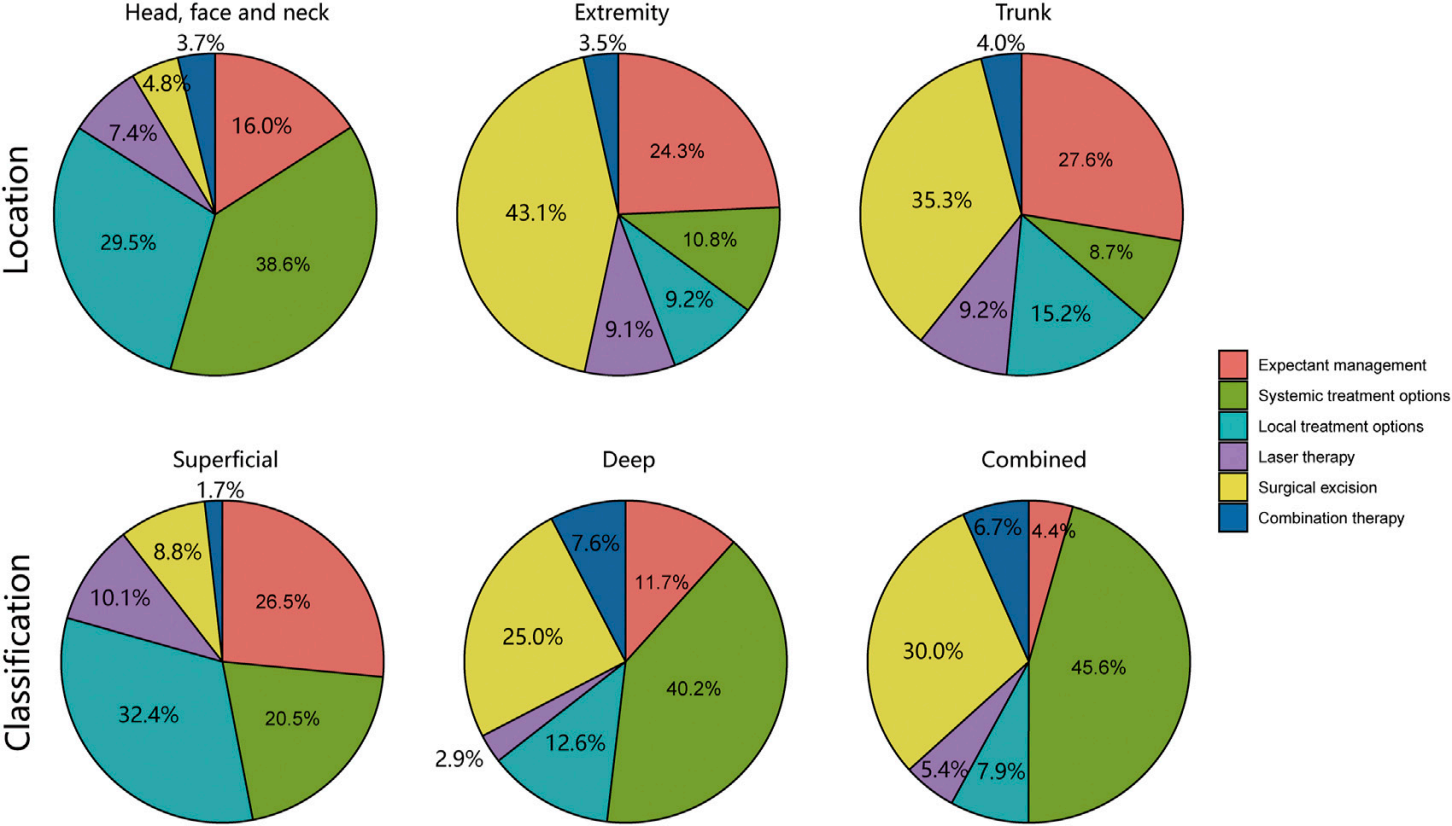
Analysis of treatment options based on location and classification of infantile hemangiomas lesions.

**FIGURE 5:**
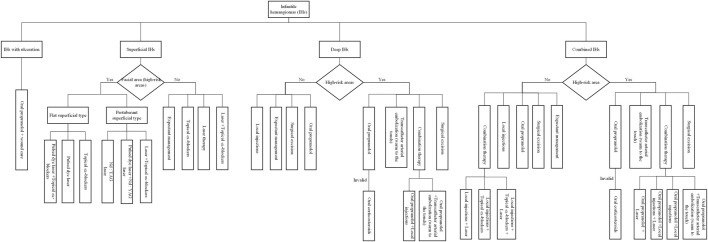
Suggested protocol for the management of infantile hemangiomas lesions.

As illustrated in Figure 4, our analysis delved into the choice of treatment based on the location and classification of infantile hemangiomas lesions. To enhance the clarity surrounding the rationale of diverse treatment choices, we conducted a comprehensive analysis encompassing the cure rate, incidence of adverse reactions, and the time cost associated with various treatment options, as presented in Table 2. Subsequently, we developed an individualized protocol, meticulously distilled in Figure 5, which encapsulates the wealth of experience our department has amassed over 22 years in managing infantile hemangiomas.

**TABLE 2:** The cure rate, incidence of adverse reactions and time cost of different treatment options. IHs infantile hemangiomas, M months.

	Expectant management (*n* = 5253)	Systemic treatment options (*n* = 8051)	Local treatment options (*n* = 6535)	Laser therapy (*n* = 2201)	Surgical excision (*n* = 4445)	Combination therapy (*n* = 1028)
Mainly for the classification of IHs	Superficial	Deep or combined	Combined	Superficial	Deep or combined	Deep or combined
IH evolution						
Color	86.2%	92.4%	88.5%	96.4%	100%	95.3%
Diameter	89.4%	86.3%	83.5%	82.3%	100%	94.5%
Deepness	92.3%	93.6%	86.4%	81.4%	100%	93.7%
Regression						
Partial	87.5%	88.3%	94.6%	92.5%	0.0%	72.8%
Total	12.5%	11.7%	5.4%	7.5%	100%	27.2%
Relapse	0.0%	3.6%	4.3%	5.3%	2.6%	3.5%
Adverse reactions	0.0%	11.5%	9.8%	13.4%	4.2%	5.3%
Mean treatment duration M)	24.2 (±6.5)	9.8 (±3.6)	6.5 (±2.6)	4.2 (±2.2)	1.2 (±0.8)	3.6 (±1.7)

### 3.5 Sequelae

All the sequelae events were documented during the treatment and follow-up. In this study, diagnoses were based on clinical manifestation and physical examination. None of those patients underwent skin biopsy. Telangiectasia, fibro-fatty tissue, and pigment change were the most frequently mentioned sequelae. Other skin sequelae identified included scar and redundant skin (Table 3).

**TABLE 3:** Sequelae of each treatment options.

Sequelae	Value (%)					
	Expectant management (*n* = 5253) (%)	Systemic treatment options (*n* = 7874) (%)	Local treatment (*n* = 3292) (%)	Laser therapy (*n* = 1621) (%)	Surgical excision (*n* = 5254) (%)	Combination therapy (*n* = 1027) (%)
Telangiectasia	14.0	42.7	8.5	14.3	7.8	26.0
fibro-fatty tissue	20.9	22.5	5.3	4.4	0.0	5.6
Hypopigmentation	3.0	5.6	14.4	24.0	0.0	16.7
Hyperpigmentation	15.9	18.6	16.5	18.0	0.0	14.4
Scar	14.8	17.6	11.3	12.5	100.0	8.9
Redundant skin	15.4	5.6	5.6	4.3	0.0	3.8

## 4 Discussion

In alignment with prior research, we identified a higher incidence of IHs in females, predominantly within the head, face, and neck region. The majority of IHs in our cohort were of the superficial type. Notably, our data demonstrated a consistent surge in IHs incidence over the observed years, a trend that mirrors the findings of an earlier study (Grzesik and Wu, 2017). This increasing pattern in IHs prevalence may be related to the escalated incidence of associated high-risk factors, such as advanced maternal age in pregnancy and premature births, in recent times.

Complex IHs account for about 12% of cases in pediatric centers, with 10%–25% of these associated with the complication of ulceration (Chamlin et al., 2007). The likelihood of ulceration depends on the tumor’s size, location, and type, with larger, superficial, and segmental tumors at a greater risk (Léauté-Labrèze et al., 2017). Although the pathogenesis of IHs ulceration remains partially obscure, the proliferation of IHs tissue plays a crucial role. Therefore, proactive measures to suppress IHs proliferation could decrease ulceration instances. Our study reveals a decrease in ulceration incidence from 22.3% to 10.0%. This decline is more evident when comparing the pre-propranolol era (21.1% ulceration) with the propranolol era (12.6% ulceration). This trend can be attributed to various factors, primarily the use of beta-blockers to control IHs proliferation. As a primary treatment for IHs, beta-blockers have allowed more patients to receive effective treatment, likely leading to the observed reduction in ulceration. The development of various treatment methods has also enabled more timely and appropriate interventions. Additionally, the introduction of guidelines and consensus statements for managing IHs has greatly improved patient outcomes, contributing to the lower ulceration rates.

After the diagnosis of IHs, comprehensive treatment considerations are warranted. Most IHs progress naturally and do not need active treatment, but consistent follow-up is necessary. Traditionally, expectant management, aligning with their spontaneous involution, was primarily employed for IHs, and intervention rates varied between 10% and 38% (Haggstrom et al., 2006b). However, with improved understanding and treatments, more cases require timely intervention. Our study observed a decline in expectant management rates, from 32.9% to 12.4%. The strategy is mainly used for infants with superficial or combined IHs, previously reported to naturally regress in about 80% of cases (Bartoshesky et al., 1978). But our clinical experience suggests a lower natural regression rate, between 15% and 25%. Moreover, post-regression complications such as scarring, pigmentation, and telangiectasia can affect a child’s wellbeing. Thus, physicians need to educate families about the characteristics of IHs, potential post-regression issues, and possible disease progression. A tumor ultrasound during the initial visit can help identify the optimal treatment timeframe (Menapace et al., 2016). Tollefson and Frieden (2012) suggested an “optimal recommended age” for treating IHs, recommending intervention around the fourth week after occurrence due to the rapid tumor growth during this period.

Systemic treatment is regarded as the first-line therapy for complicated IHs. Since Zarem and Edgerton (1967) first introduced corticosteroids for treating IHs in 1963, it remained the preferred choice for many years. However, in 2008, propranolol, initially given to two infants for cardiac reasons, showed promising results for IHs (Léauté-Labrèze et al., 2008). This led to a shift from corticosteroids to beta-blockers, particularly propranolol, due to its efficacy and tolerability, especially during the proliferative phase (Yenamandra et al., 2023). Our department, which used corticosteroids as the primary therapy, also transitioned to propranolol since 2008, relegating corticosteroids to a secondary role, used when beta-blocker therapy is contraindicated or ineffective. From 2008, 349 patients received systemic corticosteroid treatment under these circumstances. We implemented a “step dosage” regimen for propranolol to regulate usage and minimize complications. Initially, propranolol is administered twice daily, starting at 0.5 mg/kg on the first day, increased to 1.0 mg/kg on the second day, and finally to 2 mg/kg on the third day. For infants under 1 month or those with a history of preterm birth and low body weight, the maximum dose is maintained at 0.5–1.0 mg/kg.

Local treatments are often used for managing small or superficial IHs. Intralesional injections, including anti-tumor drugs like pingyangmycin, corticosteroids like dexamethasone, and vascular sclerosants like lauromacrogol, are simple, effective, and help minimize residual lesions (Hou et al., 2011; Chai et al., 2019). In our research, pingyangmycin and lauromacrogol were predominantly used for intratumor injections, particularly for IHs on the trunk and extremities. They’re less used for facial IHs due to complication risks like ulceration and scarring. Topical timolol is another effective local treatment, especially for superficial IHs. It has been recognized as a safe and effective treatment, particularly during IHs’ proliferative phase and when applied to small, superficial IHs (Chan et al., 2013). In our department, topical timolol is used as supplementary treatment during the observation period for low-risk IHs.

Laser therapy has long been a traditional method for treating IHs. As early as 1989, Glassberg et al. (1989) and colleagues reported the use of a pulsed dye laser (PDL) for large, superficial IHs. By 2002, Batta et al. (2002) and others demonstrated the effectiveness of PDL in uncomplicated IHs. Despite an initial lack of significant difference in tumor size reduction compared to the control group, PDL led to improved lesion clearance rates a year later. Various lasers, including CO2, Nd:YAG, PDL, argon ion, and flash pump PDL, can now be used for IHs, with PDL showing the highest effectiveness. Many hospitals have achieved favorable clinical outcomes using various laser therapies (Sebaratnam et al., 2021). Our department, since introducing laser tools, has increasingly utilized Nd:YAG and PDL, reinforcing their effectiveness and acceptance in IHs treatment.

Surgical treatment is a common approach for IHs unresponsive to drug therapies or physical interventions (Satterfield and Chambers, 2019), although it carries risks like peripheral tissue injury, anesthesia complications, intraoperative bleeding, postoperative scarring, infections, and potential skin grafting needs (Couto et al., 2012). Surgery is generally performed during the stable or regressive phase of IHs, with method choice depending on factors like lesion location, tumor size and depth, and the resulting skin defect after resection. The surgery aims to remove the tumor, correct local deformities, and address the physical and psychosocial impacts of IHs. It is routine to pathologically examine resected tumors to understand their characteristics and to check for residual tumor. Although propranolol has reduced the need for surgery in our department, from 25.0% to 8.5%, it is not always possible to completely avoid surgical intervention for effective symptom management. Consequently, surgery remains a vital element in the comprehensive management of IHs.

Ongoing advancements have highlighted combination therapy as a key strategy for managing complex IHs, with growing research indicating improved effectiveness from various combination protocols. Combining sclerotherapy with PDL and Nd:YAG laser treatments has demonstrated excellent efficacy, reducing adverse reactions and treatment duration (Lin et al., 2019). The adaptability of laser penetration to specific IH types and depths allows for personalized treatment. Oral propranolol combined with laser therapy, or topical *β*-receptor blockers combined with lasers, has shown increased effectiveness over monotherapy, even in children under 6 months (Fei et al., 2020). While higher doses and longer durations of propranolol yield higher success rates, they also increase adverse reactions.

Combining propranolol with PDL lowers the incidence of adverse reactions. Therefore, combining *β*-blockers and lasers may be the first-line treatment for IHs (Fei et al., 2020). Chelleri et al. (2020) found that 41.4% of patients with IHs who received treatment still had residual lesions. These residual lesions are still effectively treated with lasers and topical timolol, so laser therapy and topical timolol are potential effective treatments to reduce the incidence of residual lesions. Qiao et al. (2020) believe that the combined treatment of topical timolol and oral propranolol for IHs may be more effective than any single treatment strategy. In our study, we similarly employed combination therapy as the main strategy for managing combined IHs in recent years. This therapeutic approach has received increased recognition from our team and appears to be a promising pathway for future treatment improvements.

In this study, we have dissected and encapsulated our department’s 22 years of experience in managing IHs, leading to a series of pertinent treatment procedure. We hope that this protocol will serve as a valuable resource for the individualized treatment of IHs, shedding light on the most effective strategies to manage this complex condition. The ultimate goal is to offer each child the highest chance of complete recovery with the least possible adverse effects. Our study comes with several significant limitations which we acknowledge. For instance, its retrospective nature restricts the data collection scope and limits the kind of inferences we can draw from our findings. Also, as the study was conducted in a single-center hospital, there’s a likely chance of selection bias, possibly leading to the underrepresentation of smaller IHs. Despite these limitations, to the best of our knowledge, this is the largest Asian study describing the evolution of IHs management strategies, and it provides insights from a 22-year experience.

## 5 Conclusion

In summary, this study demonstrates that treatment for IHs has evolved over recent decades, transitioning from more invasive to less invasive medical interventions. Furthermore, an individualized protocol established from our previous treatment experience, which may hold the potential to refine the current treatment strategies for IHs.

## Data Availability

The original contributions presented in the study are included in the article/Supplementary material, further inquiries can be directed to the corresponding author.

## Abbreviations

IHs, infantile hemangiomas; IQR, interquartile range; LA, lauromacrogol; ND:YAG, neodymium-doped yttrium aluminium garnet; PDL, pulsed dye laser; PY, pingyangmycin; US, ultrasound.

## References

[B1] Auger N. Fraser W. D. Arbour L. Healy-Profitós J. Drolet B. A. (2017). Pre-eclampsia and risk of infantile haemangioma. Br. J. Dermatol 176 (2), 371–377. 10.1111/bjd.14958 27514292

[B2] Bartoshesky L. E. Bull M. Feingold M. (1978). Corticosteroid treatment of cutaneous hemangiomas: how effective? A report on 24 children. Clin. Pediatr. (Phila). 17 (8), 629–638. 10.1177/000992287801700807 78785

[B3] Baselga E. Roe E. Coulie J. Muñoz F. Z. Boon L. M. McCuaig C. (2016). Risk factors for degree and type of sequelae after involution of untreated hemangiomas of infancy. JAMA Dermatol 152 (11), 1239–1243. 10.1001/jamadermatol.2016.2905 27540637

[B4] Batta K. Goodyear H. M. Moss C. Williams H. C. Hiller L. Waters R. (2002). Randomised controlled study of early pulsed dye laser treatment of uncomplicated childhood haemangiomas: results of a 1-year analysis. Lancet 360 (9332), 521–527. 10.1016/S0140-6736(02)09741-6 12241656

[B5] Bauland C. G. Lüning T. H. Smit J. M. Zeebregts C. J. Spauwen P. H. M. (2011). Untreated hemangiomas: growth pattern and residual lesions. Plast. Reconstr. Surg. 127 (4), 1643–1648. 10.1097/PRS.0b013e318208d2ac 21460670

[B6] Chai Y. Zhou Z. Song J. Lv R. Xu G. Bi J. (2019). Safety of intralesional injection of lauromacrogol combined with triamcinolone for infantile hemangiomas. J. Dermatol 46 (9), 770–776. 10.1111/1346-8138.14992 31270853

[B7] Chamlin S. L. Haggstrom A. N. Drolet B. A. Baselga E. Frieden I. J. Garzon M. C. (2007). Multicenter prospective study of ulcerated hemangiomas. J. Pediatr. 151 (6):684–689. 10.1016/j.jpeds.2007.04.055 18035154

[B8] Chan H. McKay C. Adams S. Wargon O. (2013). RCT of timolol maleate gel for superficial infantile hemangiomas in 5- to 24-week-olds. Pediatrics 131 (6), e1739–e1747. 10.1542/peds.2012-3828 23650294

[B9] Chang L. C. Haggstrom A. N. Drolet B. A. Baselga E. Chamlin S. L. Garzon M. C. (2008). Growth characteristics of infantile hemangiomas: implications for management. Pediatrics 122 (2), 360–367. 10.1542/peds.2007-2767 18676554

[B10] Chelleri C. Monzani N. A. Gelmetti C. Milani G. P. Fossali E. F. Galeone C. (2020). Residual lesions after pharmacological and dye-laser treatment of infantile hemangiomas: critical review of 432 cases. Lasers Surg. Med. 52 (7), 597–603. 10.1002/lsm.23205 31828809

[B11] Chen T. S. Eichenfield L. F. Friedlander S. F. (2013). Infantile hemangiomas: an update on pathogenesis and therapy. Pediatrics 131 (1), 99–108. 10.1542/peds.2012-1128 23266916

[B12] Colonna V. Resta L. Napoli A. Bonifazi E. (2010). Placental hypoxia and neonatal haemangioma: clinical and histological observations. Br. J. Dermatol 162 (1), 208–209. 10.1111/j.1365-2133.2009.09493.x 19863512

[B13] Couto R. A. Maclellan R. A. Zurakowski D. Greene A. K. (2012). Infantile hemangioma: clinical assessment of the involuting phase and implications for management. Plast. Reconstr. Surg. 130 (3), 619–624. 10.1097/PRS.0b013e31825dc129 22575857

[B14] Darrow D. H. Greene A. K. Mancini A. J. Nopper A. J. SECTION ON DERMATOLOGY, SECTION ON OTOLARYNGOLOGY–HEAD AND NECK SURGERY, and SECTION ON PLASTIC SURGERY (2015). Diagnosis and management of infantile hemangioma. Pediatrics 136 (4), e1060–e1104. 10.1542/peds.2015-2485 26416931

[B15] de Jong S. Itinteang T. Withers A. H. Davis P. F. Tan S. T. (2016). Does hypoxia play a role in infantile hemangioma? Arch. Dermatol Res. 308 (4), 219–227. 10.1007/s00403-016-1635-x 26940670

[B16] Drolet B. A. Frieden I. J. (2010). Characteristics of infantile hemangiomas as clues to pathogenesis: does hypoxia connect the dots? Arch. Dermatol 146 (11), 1295–1299. 10.1001/archdermatol.2010.1295 21079070

[B17] Fei Q. Lin Y. Chen X. (2020). Treatments for infantile Hemangioma: a systematic review and network meta-analysis. EClinicalMedicine 26, 100506. 10.1016/j.eclinm.2020.100506 33089121 PMC7565185

[B18] Fu R. Zou Y. Wu Z. Jin P. Cheng J. Bai H. (2023). Safety of oral propranolol for neonates with problematic infantile hemangioma: a retrospective study in an Asian population. Sci. Rep. 13 (1), 5956. 10.1038/s41598-023-33105-2 37046020 PMC10097822

[B19] Glassberg E. Lask G. Rabinowitz L. G. Tunnessen W. W. (1989). Capillary hemangiomas: case study of a novel laser treatment and a review of therapeutic options. J. Dermatol Surg. Oncol. 15 (11), 1214–1223. 10.1111/j.1524-4725.1989.tb03235.x 2808890

[B20] Goelz R. Poets C. F. (2015). Incidence and treatment of infantile haemangioma in preterm infants. Arch. Dis. Child. Fetal Neonatal Ed. 100 (1), F85–F91. 10.1136/archdischild-2014-306197 25352093

[B21] Gomes R. Salazar L. Fraga C. Correia M. R. Barbosa-Sequeira J. Fernandes A. (2023). Management of infantile hemangiomas-experience of a tertiary hospital. Eur. J. Pediatr. 182 (4), 1611–1618. 10.1007/s00431-023-04827-2 36705724

[B22] Gong X. Qiu T. Feng L. Yang K. Dai S. Zhou J. (2022). Maternal and perinatal risk factors for infantile hemangioma: a matched case-control study with a large Sample size. Dermatol Ther. (Heidelb) 12 (7), 1659–1670. 10.1007/s13555-022-00756-4 35751738 PMC9276869

[B23] Grzesik P. Wu J. K. (2017). Current perspectives on the optimal management of infantile hemangioma. Pediatr. Health Med. Ther. 8, 107–116. 10.2147/PHMT.S115528 PMC5774589 29388636

[B24] Haggstrom A. N. Drolet B. A. Baselga E. Chamlin S. L. Garzon M. C. Horii K. A. (2006b). Prospective study of infantile hemangiomas: clinical characteristics predicting complications and treatment. Pediatrics 118 (3), 882–887. 10.1542/peds.2006-0413 16950977

[B25] Haggstrom A. N. Lammer E. J. Schneider R. A. Marcucio R. Frieden I. J. (2006a). Patterns of infantile hemangiomas: new clues to hemangioma pathogenesis and embryonic facial development. Pediatrics 117 (3), 698–703. 10.1542/peds.2005-1092 16510649

[B26] Hou J. Wang M. Tang H. Wang Y. Huang H. (2011). Pingyangmycin serotherapy for infantile hemangiomas in oral and maxillofacial regions: an evaluation of 66 consecutive patients. Int. J. Oral Maxillofac. Surg. 40 (11), 1246–1251. 10.1016/j.ijom.2011.07.906 21893396

[B27] Hunjan M. K. Schoch J. J. Anderson K. R. Lohse C. M. Marnach M. L. Hand J. L. (2017). Prenatal risk factors for infantile hemangioma development. J. Invest. Dermatol 137 (4), 954–957. 10.1016/j.jid.2016.10.047 27940221 PMC6309253

[B28] Léauté-Labrèze C. Dumas de la Roque E. Hubiche T. Boralevi F. Thambo J. B. Taïeb A. (2008). Propranolol for severe hemangiomas of infancy. N. Engl. J. Med. 358 (24), 2649–2651. 10.1056/NEJMc0708819 18550886

[B29] Léauté-Labrèze C. Harper J. I. Hoeger P. H. (2017). Infantile haemangioma. Lancet 390 (10089), 85–94. 10.1016/S0140-6736(16)00645-0 28089471

[B30] Léauté-Labrèze C. Hoeger P. Mazereeuw-Hautier J. Guibaud L. Baselga E. Posiunas G. (2015). A randomized, controlled trial of oral propranolol in infantile hemangioma. N. Engl. J. Med. 372 (8), 735–746. 10.1056/NEJMoa1404710 25693013

[B31] Lin L. Guo P. Cao Y. Li Q. Zhang J. Huo R. (2019). Combination of serotherapy and dual-wavelength laser in the management of infantile hemangiomas in Chinese infants. Dermatol Surg. 45 (10), 1253–1259. 10.1097/DSS.0000000000001898 30882500

[B32] Macca L. Altavilla D. Di Bartolomeo L. Irrera N. Borgia F. Li Pomi F. (2022). Update on treatment of infantile hemangiomas: what's new in the last five years? Front. Pharmacol. 13, 879602. 10.3389/fphar.2022.879602 35721150 PMC9204338

[B33] Menapace D. Mitkov M. Towbin R. Hogeling M. (2016). The changing face of complicated infantile hemangioma treatment. Pediatr. Radiol. 46 (11), 1494–1506. 10.1007/s00247-016-3643-6 27450406

[B34] Munden A. Butschek R. Tom W. L. Marshall J. S. Poeltler D. M. Krohne S. E. (2014). Prospective study of infantile haemangiomas: incidence, clinical characteristics and association with placental anomalies. Br. J. Dermatol 170 (4), 907–913. 10.1111/bjd.12804 24641194 PMC4410180

[B35] Polites S. F. Watanabe M. Crafton T. Jenkins T. M. Alvarez-Allende C. R. Hammill A. M. (2019). Surgical resection of infantile hemangiomas following medical treatment with propranolol versus corticosteroids. J. Pediatr. Surg. 54 (4), 740–743. 10.1016/j.jpedsurg.2018.08.001 30249358

[B36] Qiao J. Lin J. Zhang D. Chen C. Yu H. (2020). Efficacy of combined topical timolol and oral propranolol for treating infantile hemangioma: a meta-analysis of randomized controlled trials. Front. Pharmacol. 11, 554847. 10.3389/fphar.2020.554847 33132908 PMC7578425

[B37] Rodríguez Bandera A. I. Sebaratnam D. F. Wargon O. Wong L. C. F. (2021). Infantile hemangioma. Part 1: epidemiology, pathogenesis, clinical presentation and assessment. J. Am. Acad. Dermatol 85 (6), 1379–1392. 10.1016/j.jaad.2021.08.019 34419524

[B38] Satterfield K. R. Chambers C. B. (2019). Current treatment and management of infantile hemangiomas. Surv. Ophthalmol. 64 (5), 608–618. 10.1016/j.survophthal.2019.02.005 30772366

[B39] Sebaratnam D. F. Rodríguez Bandera A. L. Wong L. F. Wargon O. (2021). Infantile hemangioma. Part 2: management. J. Am. Acad. Dermatol 85 (6), 1395–1404. 10.1016/j.jaad.2021.08.020 34419523

[B40] Tollefson M. M. Frieden I. J. (2012). Early growth of infantile hemangiomas: what parents' photographs tell us. Pediatrics 130 (2), e314–e320. 10.1542/peds.2011-3683 22826568

[B41] Yenamandra V. K. Khute P. Yadav D. Narayanan A. Tekumalla S. V S. (2023). Oral propranolol therapy for infantile hemangioma: long-term follow-up. Indian J. Pediatr. 90, 937–939. 10.1007/s12098-023-04586-w 37204593

[B42] You Y. Li Y. Xiao Y. Zhang J. (2021). Propranolol vs. steroids in the treatment of infantile hemangiomas: a meta-analysis. Mol. Clin. Oncol. 15 (2), 156. 10.3892/mco.2021.2318 34178327 PMC8220686

[B43] Zarem H. A. Edgerton M. T. (1967). Induced resolution of cavernous hemangiomas following prednisolone therapy. Plast. Reconstr. Surg. 39 (1), 76–83. 10.1097/00006534-196701000-00010 6018814

